# Proliferative Effects of Histamine on Primary Human Pterygium Fibroblasts

**DOI:** 10.1155/2016/9862496

**Published:** 2016-10-30

**Authors:** Zhenwei Qin, Qiuli Fu, Lifang Zhang, Houfa Yin, Xiuming Jin, Qiaomei Tang, Danni Lyu, Ke Yao

**Affiliations:** ^1^Eye Center of the 2nd Affiliated Hospital, Medical College of Zhejiang University, Hangzhou, Zhejiang, China; ^2^Zhejiang Provincial Key Lab of Ophthalmology, Hangzhou, Zhejiang, China

## Abstract

*Purpose*. It has been confirmed that inflammatory cytokines are involved in the progression of pterygium. Histamine can enhance proliferation and migration of many cells. Therefore, we intend to investigate the proliferative and migratory effects of histamine on primary culture of human pterygium fibroblasts (HPFs).* Methods*. Pterygium and conjunctiva samples were obtained from surgery, and toluidine blue staining was used to identify mast cells. 3-[4, 5-Dimethylthiazol-2-yl]-2,5-diphenyltetrazolium bromide (MTT) was performed to evaluate the proliferative rate of HPFs and human conjunctival fibroblasts (HCFs); ki67 expression was also measured by immunofluorescence analysis. Histamine receptor-1 (H1R) antagonist (Diphenhydramine Hydrochloride) and histamine receptor-2 (H2R) antagonist (Nizatidine) were added to figure out which receptor was involved. Wound healing model was used to evaluate the migratory ability of HPFs.* Results*. The numbers of total mast cells and degranulated mast cells were both higher in pterygium than in conjunctiva. Histamine had a proliferative effect on both HPFs and HCFs, the effective concentration (10 *μ*mol/L) on HPFs was lower than on HCFs (100 *μ*mol/L), and the effect could be blocked by H1R antagonist. Histamine showed no migratory effect on HPFs.* Conclusion*. Histamine may play an important role in the proliferation of HPFs and act through H1R.

## 1. Introduction

Pterygium is a benign, chronic overgrowth of fibrovascular conjunctiva that lies over the nasal or temporal cornea. It can cause visual impairment, astigmatism, and cosmetic issues and ultimately affect the quality of life. Although pterygium is a benign disease, it is also considered to be a neoplastic-like disorder with its uncontrolled proliferation, migration, angiogenesis, and recurrence [1]. It has been identified that the expression of genes associated with cell proliferation and angiogenesis, such as PCNA, mutant p53, MAP kinase signaling pathway, matrix metalloproteinases, and VEGFA, is higher in the pterygium than normal conjunctiva tissues [2–6]. Such properties make pterygium similar to a tumor in some ways.

It has been estimated that many issues such as genetic changes, environmental influences, and HPV infection are involved in the progression of pterygium [7]. Also, various chronic inflammatory stimuli, such as ultraviolet irradiation, sawdust exposure, and dry eye disease, have been confirmed to be related to pterygium formation by epidemiologic studies [8, 9]. Multiple proinflammatory genes, such as nuclear factor-kappa beta (NF-*κ*B), IL-1 beta, TNF-alpha, the receptor for advanced glycation end-products (RAGE), S100A8/A9, and other cytokines, have been reported to participate in the progression of pterygium [10–13]. Therefore, inflammatory cytokines were considered to play an important role in the development of pterygium.

Mainly released by mast cells, histamine is an important inflammatory cytokine confirmed to be a key mediator in allergic ocular diseases, such as vernal keratoconjunctivitis and allergic conjunctivitis [14, 15]. Histamines were also found in increased concentrations in the tears of such patients. Moreover, histamine was proved to stimulate cell behavior in multiple cell lines derived from human neoplasia, such as colon carcinoma, lung cancer cells, astrocytoma, and other cancer cells, through one or more histamine receptors [16–19]. There was evidence showed that histamine concentration, as well as mast cell number, was greater in human breast cancer and colorectal cancer than in normal tissue [19, 20], suggesting the important role histamine plays not only in the process of allergic diseases, but also in modulating cell proliferation and migration. Most importantly, histamine has proved to be a promotive factor to the proliferation and migration of fibroblast derived from conjunctival tissue [21]. Besides, functional histamine receptor was proved to be expressed on pterygium [22].

The aim of this study was to evaluate the effect of histamine on human pterygium fibroblasts and, based on the reasons outlined above, we hypothesized that histamine may play a promotive role in the progression of pterygium.

## 2. Materials and Methods

### 2.1. Isolation and Expansion of Human Pterygium Fibroblasts

Five different human pterygium samples and three human conjunctiva samples were obtained by surgical means from patients who provided informed written consent. The diagnosis of pterygium was entirely clinical with no pathological evidence. Pterygium and conjunctiva samples were put in culture medium [Dulbecco's modified Eagle medium (DMEM; Gibco Life Technologies, Karlsruhe, Germany) supplemented with 10% fetal bovine serum (FBS; Gibco Life Technologies), 100 U/mL penicillin, and 100 g/mL streptomycin (Gibco Life Technologies)] just after surgery and transported on ice to our lab. Samples were washed with phosphate-buffered saline (PBS; Gibco Life Technologies) three times, cut into small pieces (about 2 mm × 2 mm size), and incubated in trypsin-EDTA (0.25%, Gibco Life Technologies) at 37°C for 15 minutes; the solution was then filtered using a 70 *μ*m cell strainer (BD Falcon, NJ, USA) and centrifuged at 200 ×g for 5 minutes, and the sediment was resuspended with culture medium and incubated at 37°C in 5% CO_2_ in a humidified atmosphere. The medium was changed every 2 days thereafter. Cells between third and ninth passage were used for experiments.

### 2.2. 3-[4, 5-Dimethylthiazol-2-yl]-2,5-diphenyltetrazolium Bromide (MTT) Assay

HPFs or HCFs were seeded in 96-well culture plates at a concentration of 2.5 k cells/well in culture medium and incubated for 24 hours. These cells were then treated with different concentrations of serum or histamine for another 48 hours. HR antagonists were added to cells 4 hours early before treatment of histamine to figure out which receptor was involved. In addition, a 24-hour starving period was implemented to eliminate the effect of culture medium. Then the cells were incubated with MTT (Sigma, CO, USA) at a final concentration of 0.5 mg/mL. After 4 hours, the MTT solution was discarded and 150 *μ*L DMSO (Sigma, CO, USA) was added to dissolve the formazan precipitate by shaking the plates for 10 minutes at mild speed on an orbital shaker. Microplate readers (Bio-Rad, Munich, Germany) were used to read the absorbance of each well at the wavelength of 540 nm.

### 2.3. Immunofluorescence

HPFs were fixed with 4% paraformaldehyde (Sigma, CO, USA) for 15 minutes and permeabilized with 0.3% Triton-X100 (Sigma, CO, USA) in PBS for 15 minutes after being treated with 0%, 5%, and 10% FBS for 48 hours. Then the HPFs were incubated with primary rabbit polyclonal anti-ki67 antibody (1 : 100 dilutions, Thermo Scientific, IL, USA) overnight at 4°C. The HPFs were then incubated for 1 hour with secondary goat anti-rabbit IgG (H+L) (1 : 1000 dilutions, Alexa Fluor 555, OR, USA). Nuclei were stained with 2-(4-amidinophenyl)-6-indolecarbamidine dihydrochloride (Sigma, CO, USA) at a concentration of 1 *μ*g/mL. The HPFs were analyzed with a fluorescent microscope (Olympus DP72, Japan). The ki67 positive rate was calculated 15 times by random fields of microscope.

### 2.4. Toluidine Blue Staining

Three pairs of conjunctiva and pterygium tissues were collected after surgery and immediately fixed in 10% formalin overnight in 4°C. Paraffin-embedded samples were sectioned at 4 *μ*m thickness, and the sections were deparaffinized in xylene and rehydrated in a graded series of ethanol solutions. The sections were stained with 0.5% toluidine blue (Sigma, CO, USA) working solution for 10 minutes and washed three times with distilled water. The sections were differentiated by 0.5% acetic acid (Sigma, CO, USA) solution until nuclear and cytoplasmic granules were clearly visualized. Sections were then cleared in xylene and mounted with mounting medium. The mast cells were counted 15 times by random fields of microscope for each sample.

### 2.5. Real-Time Quantitative Polymerase Chain Reaction (RT-qPCR)

Total RNA was isolated (Trizol Reagent, Invitrogen, CA, USA) and reverse transcribed (Promega, WI, USA) according to the manufacturer's protocol. The SYBR Premix Ex Taq (TaKaRa, Shiga, Japan) was used for real-time quantitative PCR according to the manufacturer's protocol on a 7500 Fast Real-Time PCR System (ABI, CA, USA). The primers were shown in Table 1. Glyceraldehyde-3-phosphate dehydrogenase (GAPDH) was used as an endogenous reference. The quantification cycle (Ct) was obtained, and the ΔCt value was calculated with Ct_(gene)_ − Ct_(GAPDH)_. ΔΔCt was calculated with ΔCt_(H1R)_ − ΔCt_(H2R)_ and then converted to 2^(−ΔΔCt)^ to get the fold-change (FC).

### 2.6. DNA Agarose Gel Electrophoresis

The electrophoresis was run using a 2% agarose gel submerged in 0.5x Tris-borate-EDTA (TBE) buffer at 0.3 V/cm for 45 minutes at room temperature. Ethidium bromide was added to the gel at a final concentration of 0.5 *μ*g/mL. The analysis was completed on a ChemiDoc MP Imaging System (Bio-Rad, Munich, Germany).

### 2.7. Wound Healing Assay

HPFs were seeded in 24-well plates. After 24 h of culture, the cell density of each well reached 90% confluency, and then the cells were scratched with a sterile 100 *μ*L pipette tip. Scratched wells were washed with PBS for three times, and mediums with or without histamine were added to wells. The wounds were photographed at 0, 8, and 24 h. The area of the remaining wound in each image was measured using the ImageJ software (National Institutes of Health, MD, USA). The data were quantified based on the area of wound at 0 h; the wound at 0 h was considered as 100%. The results were repeated for three times.

### 2.8. Statistical Analysis

All experiments were performed at least three times. Quantitative data are presented as the mean ± SEM and were analyzed by one-way analysis of variance (ANOVA). A *P* value < 0.05 was considered statistically significant.

## 3. Results

### 3.1. Serum Had a Significant Effect on the Proliferative Ability of HPFs and Was Highly Related to Its Concentration

**Figure 1:**
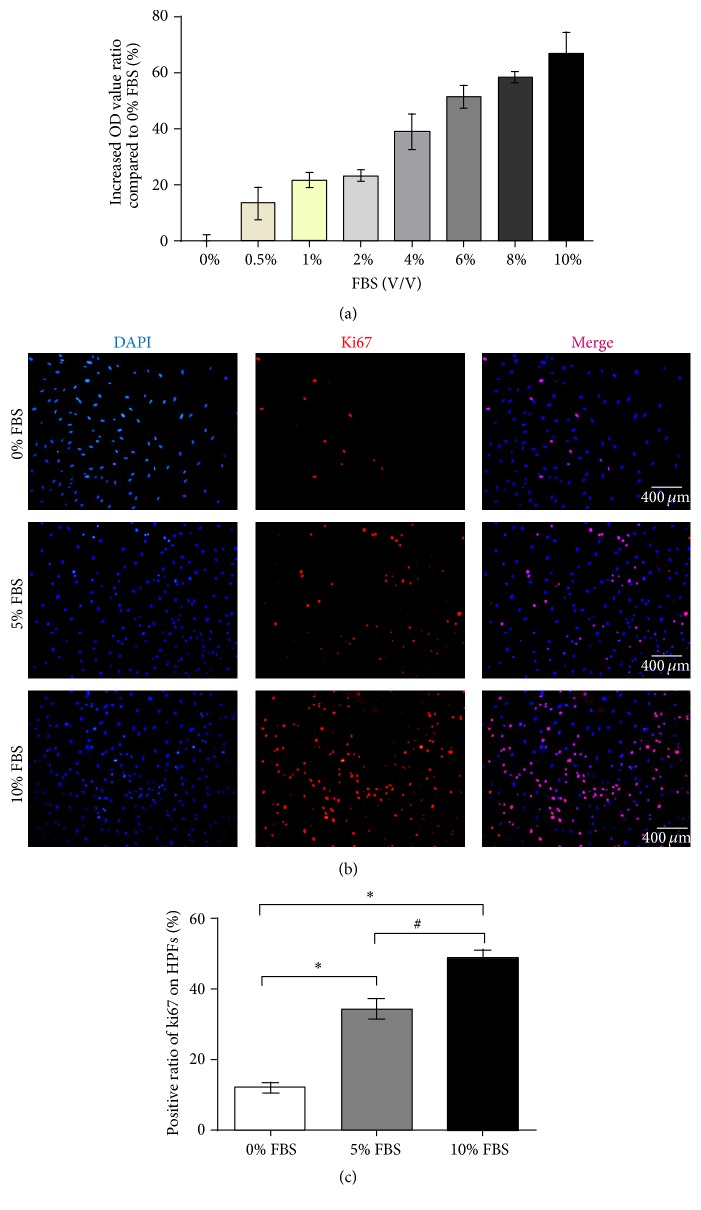
Serum had a strong proliferative effect on HPFs. (a) MTT assay showed serum had a proliferative effect on every concentration from 0.5% FBS to 10% FBS and the effect increased along with increased concentrations of serum. (b) Immunofluorescence staining of ki67 on HPFs in 0% FBS, 5% FBS, and 10% FBS. (c) The positive rate of ki67 on HPFs increased along with increased concentrations of serum. ^*∗*^ *P* < 0.05 compared to 0% FBS, ^#^ *P* < 0.05 compared to 5% FBS.

We simulated different vessel ratios of pterygium in vitro by using different concentrations of serum. MTT assay showed that all concentrations of serum have a positive effect on HPF growth, the effect started to reach a relatively stable state at 6% FBS, and the maximal effect was obtained with 10% FBS, its 66.74 ± 7.77% additional proliferation compared to serum-free control (Figure 1(a)). The expression of ki67 on HPF was also related to the concentration of serum, and the highest ki67 positive ratio was 48.74 ± 6.23% with 10% FBS culture (Figures 1(b) and 1(c)).

### 3.2. The Numbers of Total Mast Cells and Degranulated Mast Cells in Pterygium Were Both Higher Than in Conjunctiva

**Figure 2:**
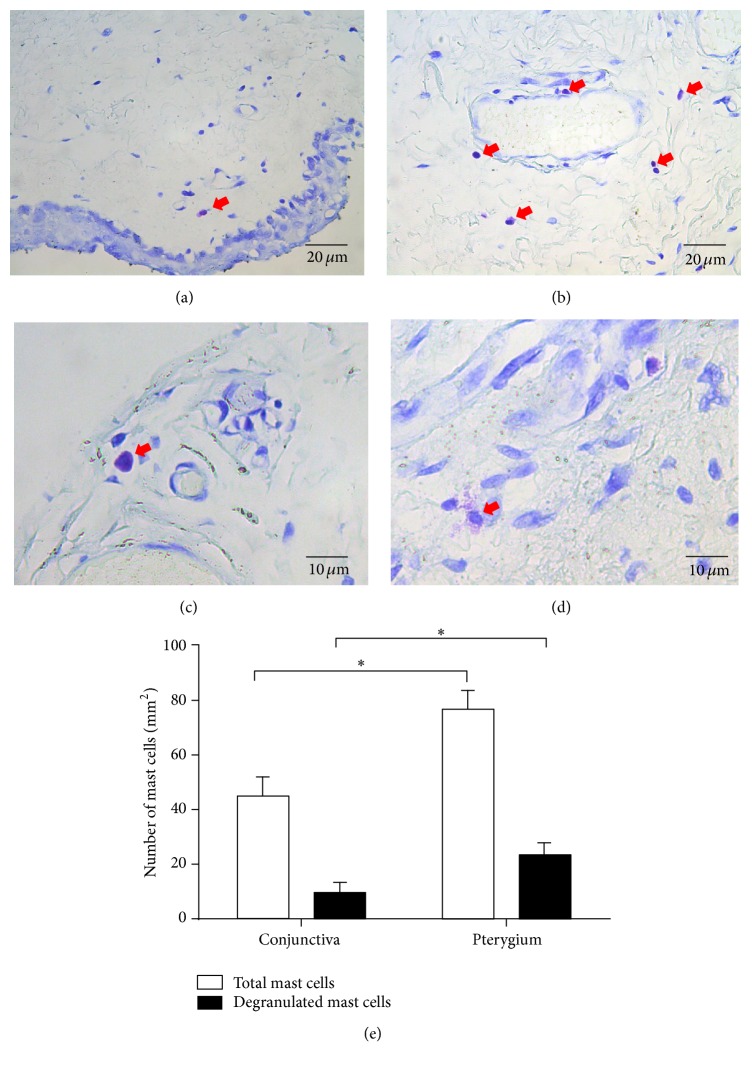
Toluidine blue staining of pterygium and conjunctiva: mast cells showed the specific violet staining. (a, b) Typical photos of toluidine blue staining of mast cells in conjunctiva (a) and pterygium (b). (c, d) Typical photos of toluidine blue staining of intact mast cells (c) and degranulated mast cells (d). (e) The numbers of total mast cells and degranulated mast cells per mm^2^ counted in 15 random fields of microscope: both the numbers were greater in pterygium than in conjunctiva. ^*∗*^ *P* < 0.05 compared to conjunctiva.

Mast cells showed a specific violet staining by toluidine blue staining. Our results revealed that both pterygium and conjunctiva showed the expression of mast cells (Figures 2(a)–2(d)), the numbers of total mast cells and degranulated mast cells per mm^2^ were 76.79 ± 6.40 and 23.46 ± 3.69 in pterygium, and they were both more than in conjunctiva (44.79 ± 6.40 and 9.60 ± 3.20) (Figure 2(e)).

### 3.3. Histamine Had a Proliferative Effect on HPFs in Situations Both with and without Serum

**Figure 3:**
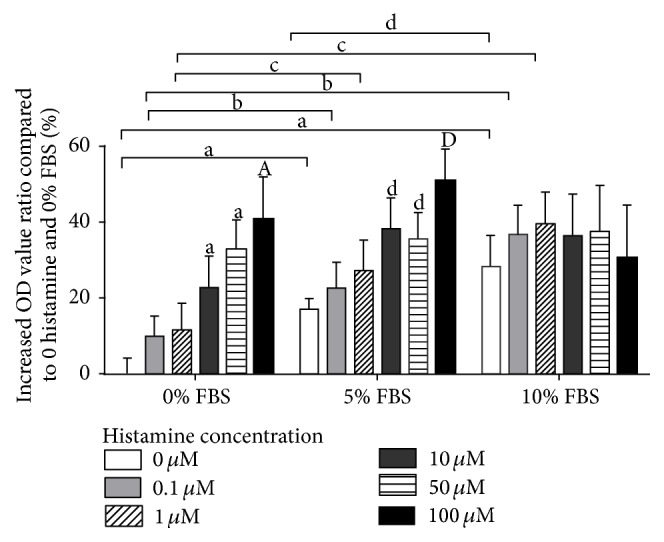
Histamine had a proliferative effect on HPFs. Histamine promoted the proliferation of HPFs with concentrations of 10 *μ*mol/L and above in the situations of 0% FBS and 5% FBS. High concentration (10–100 *μ*mol/L) of histamine could partially cover the promotive effect of serum on HPFs. ^a^ *P* < 0.05 compared to 0 histamine and 0% FBS, ^A^ *P* < 0.01 compared to 0 histamine and 0% FBS, ^b^ *P* < 0.05 compared to 0.1 *μ*mol/L histamine and 0% FBS, ^c^ *P* < 0.05 compared to 1 *μ*mol/L histamine and 0% FBS, ^d^ *P* < 0.05 compared to 0 histamine and 5% FBS, and ^D^ *P* < 0.01 compared to 0 histamine and 5% FBS.

Compared with drug-free control, MTT assay showed that histamine had a proliferative effect on HPF growth at concentrations above 10 *μ*mol/L when in 0% FBS and 5% FBS situation. There was an increasing proliferative effect trend, but no statistical difference among the increasing concentration of histamine. However, histamine did not show a proliferative effect in 10% FBS situation at any concentration. When in a lower concentration (0-1 *μ*mol/L) of histamine situation, FBS could promote the proliferation of HPFs, and in a higher concentration (10–100 *μ*mol/L) of histamine situation, the promotive effect of FBS was not obvious, indicating that histamine could partially compensate the influence brought by serum insufficiency (Figure 3).

### 3.4. HPFs Express H1R, H2R, and H4R and the Effect of Histamine Can Be Blocked by H1R Antagonist

**Figure 4:**
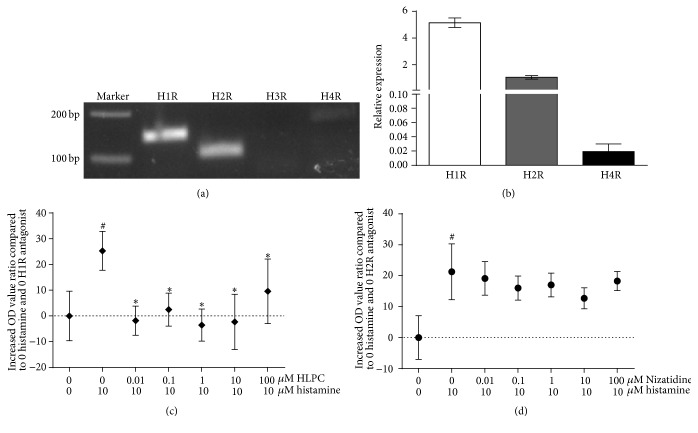
The expression and role of histamine receptors on HPFs. (a) DNA agarose gel electrophoresis showed the expression of H1R, H2R, and H4R on HPFs. (b) RT-qPCR showed that the expression of H1R was about 5-fold more than H2R and the expression of H4R was quite few. (c) MTT assay showed that H1R antagonist significantly inhibited the proliferation of HPFs induced by histamine. (d) MTT assay showed that H2R antagonist did not show the inhibition effect. ^*∗*^ *P* < 0.05 compared to incubation with 10 *μ*mol/L of histamine alone. ^#^ *P* < 0.05 compared to incubation without histamine and antagonist.

In four known histamine receptors, H1R, H2R, and H4R were confirmed to exist in HPFs by real-time qPCR and agarose gel electrophoresis (Figure 4(a)). H1R has the highest expression and H4R has the least (Figure 4(b)). The effect of histamine can be blocked by H1R antagonist Diphenhydramine Hydrochloride (HLPC) at concentrations from 0.01 to 100 *μ*mol/L (Figure 4(c)), while H2R antagonist Nizatidine did not show the antagonistic effect (Figure 4(d)), indicating that H1R was involved in the action of histamine.

### 3.5. Histamine Also Had Proliferative Effect on HCFs but at a Higher Effective Concentration than on HPFs

**Figure 5:**
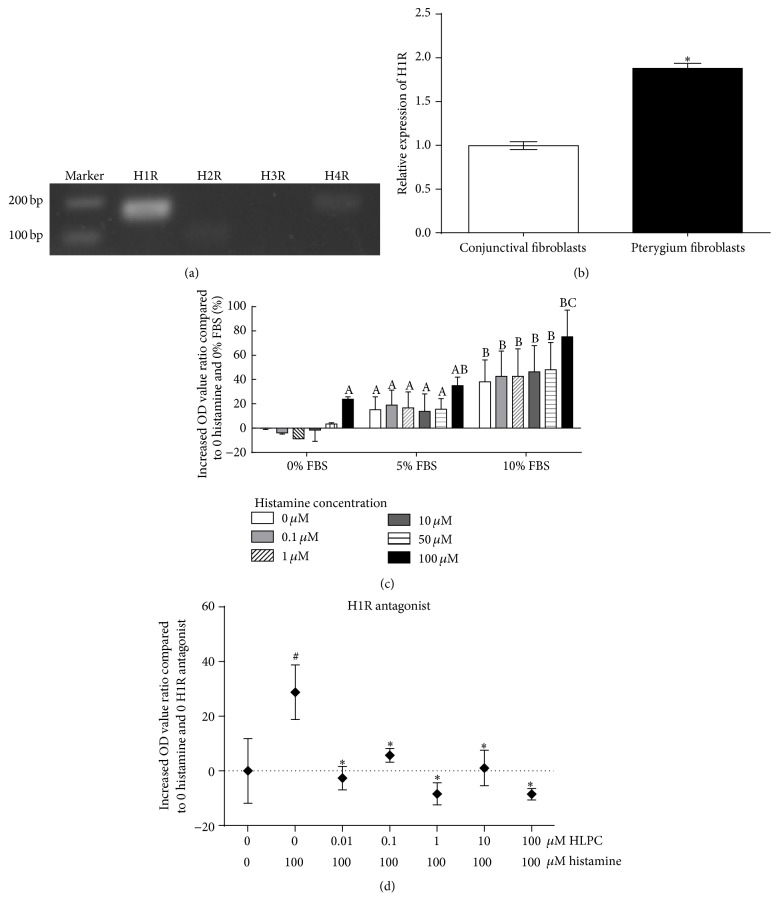
Histamine also showed proliferative effect on HCFs but at a higher effective concentration than on HPFs. (a) DNA agarose gel electrophoresis showed the expression of H1R, H2R, and H4R on HCFs, and the expression of H2R and H4R was quite few. (b) RT-qPCR showed that the expression of H1R in HCFs was only half of those in HPFs. (c) MTT assay showed that histamine had proliferative effect on HCFs in 0%, 5%, and 10% FBS situation at concentration of 100 *μ*mol/L. ^A^ *P* < 0.05 compared to 0 histamine and 0% FBS, ^B^ *P* < 0.05 compared to 0 histamine and 5% FBS, ^C^ *P* < 0.05 compared to 0 histamine and 10% FBS. (d) MTT assay showed that H1R antagonist could efficiently inhibit the proliferative effect of histamine. ^*∗*^ *P* < 0.05 compared to incubation with 100 *μ*mol/L of histamine alone. ^#^ *P* < 0.05 compared to incubation without histamine and antagonist.

H1R, H2R, and H4R were also confirmed to exist in HCFs, the expression of H2R and H4R was both quite few in HCFs, and the expression of H1R in HCFs was only half of those in HPFs (Figures 5(a) and 5(b)). According to MTT assay, histamine also had proliferative effect on HCFs but at a much higher effective concentration (100 *μ*mol/L) compared to this (10 *μ*mol/L) in HPFs. The effect could be found under 0%, 5%, and 10% FBS situation (Figure 5(c)). The proliferative effect of histamine on HCFs could be blocked efficiently by HIR antagonist (Figure 5(d)).

### 3.6. Histamine Showed No Migratory Effect on HPFs

**Figure 6:**
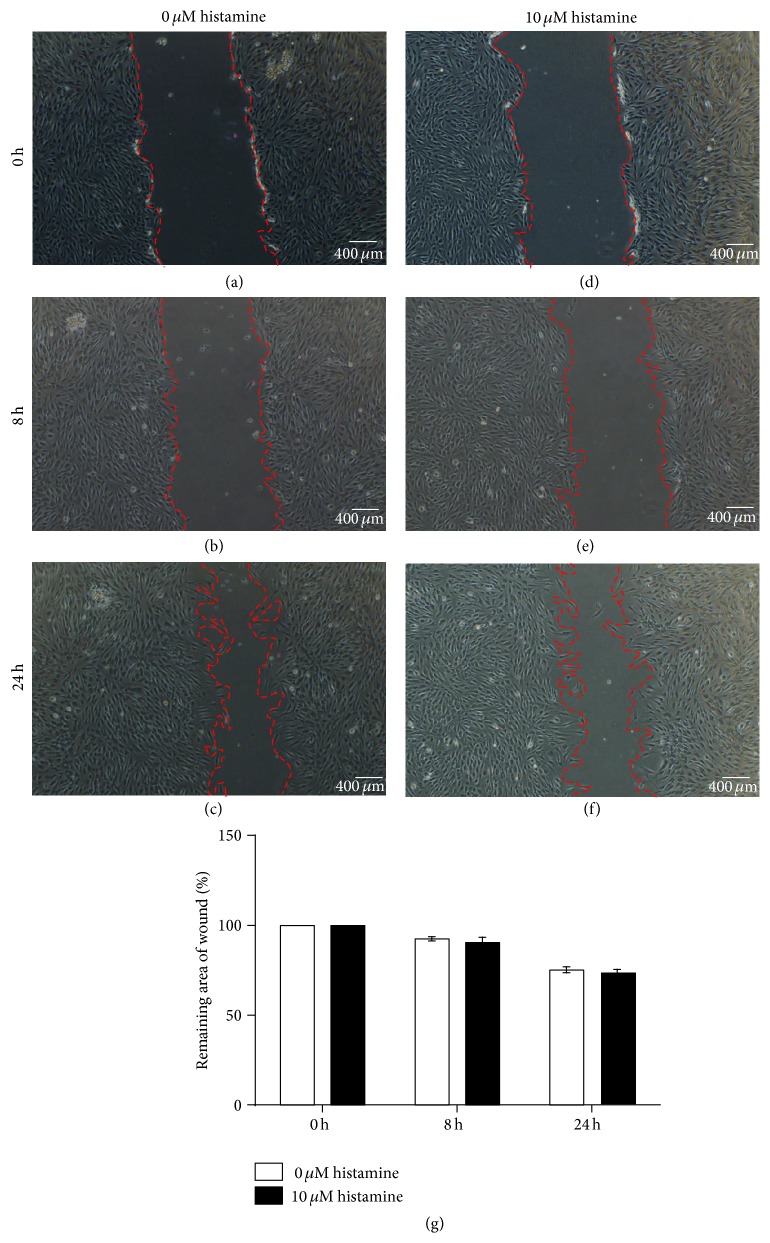
Histamine showed no migratory effect on HPFs. (a, b, c) Photos of wounds in 0 *μ*mol/L histamine at 0, 8, and 24 h. (d, e, f) Photos of wounds in 10 *μ*mol/L histamine at 0, 8, and 24 h. (g) The remaining area of wounds in 0 and 10 *μ*mol/L histamine at 0, 8, and 24 h.

We tested the migratory effect of histamine on HPF but got negative results (Figure 6), and histamine showed no migratory effect on HPFs in both with or without serum situation at concentration from 10 to 100 *μ*mol/L.

## 4. Discussion

To our knowledge, this is the first study investigating the involvement of histamine in the progression of pterygium. Our results show increased total mast cells and degranulated mast cells in pterygium and that histamine has a proliferative effect on HPFs at a much lower concentration than on HCFs. We further demonstrated that H1R, H2R, and H4R were expressed on both pterygium and conjunctiva. However, the expressions of H1R and H2R were both higher in HPFs than in HCFs. And this proliferative effect of histamine on HPFs acts mainly through H1R.

Pterygium is a benign disease with neoplastic-like features, such as local proliferation, migration, angiogenesis, and recurrence [1]. It will not cause aesthetic or visual influences unless it overtakes the cornea and keeps moving forward. Therefore, the incentives that excite or promote the proliferation and migration of pterygium were critical points in clinical prevention and treatment. Our results show the significant effect of serum on the proliferative ability of HPFs, which is consistent with the other published report [23], indicating that serum level is an important incentive related to the growth of pterygium. Some studies have shown that angiogenesis related genes and growth factors, such as EphB4, vascular endothelial growth factor (VEGF), anti-von-Willebrand factor (vWF), and Nestin, are highly expressed in pterygium and reveal the vascular content ratio as a main determining factor of the destiny of pterygium [24–26].

Our results show an increased number of mast cells in pterygium, which is consistent with the other published report [27]. An increased presence of mast cells has also been revealed in other chronic inflammatory conditions accompanied by fibrosis, such as pulmonary fibrosis, inflammatory bowel disease, peritoneal fibrosis, and oral submucous fibrosis, which indicates that mast cells and their mediators can modulate connective tissue metabolism [28–31]. It has been verified that increased levels of histamine can promote the proliferation of a variety of cancer cells, fibroblasts, neuron stem cell, and other cells [20, 21, 32]. Our results show that histamine has a proliferative effect on HPFs in 0% FBS and 5% FBS culture, and the effective concentration of histamine on HPFs was much lower than on HCFs, indicating that HPFs were easier to be triggered by histamine. We speculated that the reasons may be the higher expression of histamine receptors and the vigorous growth potential of pterygium. The latter reason may also explain the results why histamine did not show proliferative effect in 10% FBS situation since the effect would be covered under enough nutritional support. Also, our results showed that the promotive effect of serum on HPFs could be partially covered by high concentration of histamine. Pterygium is a fibrovascular conjunctiva with neoplastic-like features, and compared with our results of the proliferative effect of histamine on HPFs, it is reasonable to conclude that histamine is a supporting incentive that can excite and promote the proliferation of pterygium with a mild and moderate content ratio of vessels. It can also be a potential incentive related to the recurrence of pterygium. Also, any diseases such as vernal conjunctivitis and allergic conjunctivitis that will activate mast cells or other immune cells to release histamine can be supporting incentives in the progression of pterygium.

Histamine has four kinds of receptors (H1R, H2R, H3R, and H4R). H1R and H2R were widely expressed in a variety of tissues and immune cells, H3R has been confirmed to mainly localize in the brain, and H4R is preferentially expressed on immune cells, including mast cells [33]. Our results show that the three histamine receptors of H1R, H2R, and H4R were expressed in pterygium. In our study, only anti-H1R treatment reduced the proliferative effect induced by histamine. Histamine-induced proliferation has been shown to be mediated through H1R on many cells, including subcutaneous fibroblasts, neuron stem cells, astrocytoma, and lung cancer cells [17, 18, 31, 34]. Furthermore, H2R was also involved in histamine-induced fibroblast proliferation in many studies [17, 21, 32]. In our results, anti-H2R treatment could reduce the growth trend of HPFs, but this trend was not statistically significant, and we speculated that the lower expression of H2R on HPFs may be an important reason. We also tested the migratory effect of histamine on HPFs but got negative results.

In conclusion, our study demonstrated the proliferative effect of histamine on HPFs and the difference of this effect between HPFs and HCFs revealed histamine, and any diseases increasing histamine release, to be supporting incentives in the progression of pterygium. We provided new insight into the pathogenesis of pterygium and also offered possible site to prevent the recurrence of pterygium.

## Figures and Tables

**Table 1:** Primer sequences used in RT-qPCR.

Gene	Primer
H1R-F	CTGAGCACTATCTGCTTGGTC
H1R-R	AGGATGTTCATAGGCATGACGA
H2R-F	CAGCAAGGGCAATCATACCAC
H2R-R	GATCAGTAGCGGGAGGTAGAA
H3R-F	CACCCGAGCGGTCTCATAC
H3R-R	GGATGGCTGGTCCGTACAG
H4R-F	GGTGTGATCTCCATTCCTTTGT
H4R-R	CAAGACCCCAGTATGTTGAGTTC
GAPDH-F	ATTGCCCTCAACGACCACT
GAPDH-R	ATGAGGTCCACCACCCTGT
